# Combined deletion of *Bap1*, *Nf2*, and *Cdkn2ab* causes rapid onset of malignant mesothelioma in mice

**DOI:** 10.1084/jem.20191257

**Published:** 2020-04-09

**Authors:** Jitendra Badhai, Gaurav Kumar Pandey, Ji-Ying Song, Oscar Krijgsman, Rajith Bhaskaran, Gayathri Chandrasekaran, Min-chul Kwon, Lorenzo Bombardelli, Kim Monkhorst, Cristoforo Grasso, John Zevenhoven, Jan van der Vliet, Miranda Cozijnsen, Paul Krimpenfort, Daniel Peeper, Maarten van Lohuizen, Anton Berns

**Affiliations:** 1 Oncode Institute, Division of Molecular Genetics, The Netherlands Cancer Institute, Amsterdam, Netherlands; 2 Division of Molecular Genetics, The Netherlands Cancer Institute, Amsterdam, Netherlands; 3 Department of Experimental Animal Pathology, The Netherlands Cancer Institute, Amsterdam, Netherlands; 4 Oncode Institute, Division of Molecular Oncology and Immunology, The Netherlands Cancer Institute, Amsterdam, Netherlands; 5 Department of Pathology, The Netherlands Cancer Institute, Amsterdam, Netherlands

## Abstract

We have generated mouse models of malignant mesothelioma (MM) based upon disruption of the *Bap1*, *Nf2*, and *Cdkn2ab* tumor suppressor loci in various combinations as also frequently observed in human MM. Inactivation of all three loci in the mesothelial lining of the thoracic cavity leads to a highly aggressive MM that recapitulates the histological features and gene expression profile observed in human patients. The tumors also show a similar inflammatory phenotype. *Bap1* deletion alone does not cause MM but dramatically accelerates MM development when combined with *Nf2* and *Cdkn2ab* (hereafter BNC) disruption. The accelerated tumor development is accompanied by increased Polycomb repression and EZH2-mediated redistribution of H3K27me3 toward promoter sites with concomitant activation of PI3K and MAPK pathways. Treatment of BNC tumor–bearing mice with cisplatin and pemetrexed, the current frontline treatment, prolongs survival. This makes the autochthonous mouse model described here very well suited to explore the pathogenesis of MM and validate new treatment regimens for MM, including immunotherapy.

## Teaser

Badhai et al. describe a mouse model of mesothelioma with combined deletion of *Bap1*, *Nf2*, and *Cdkn2ab* that shows rapid onset and recapitulates human mesothelioma including its response to the standard treatment. This autochthonous model is well suited for testing cancer immunotherapies.

## Introduction

Malignant mesothelioma (MM) is a highly aggressive tumor of serosal surfaces. The frontline therapy only extends overall survival for a few months, and targeted therapies have largely failed in the clinic (Vogelzang et al., 2003). The genomic landscape of MM shows frequent inactivation of the *CDKN2AB* locus that encodes for the p16^INK4A^, p15^INK4B^, and p14^ARF^ cell cycle inhibitor proteins and the Neurofibromatosis Type 2 (*NF2*) tumor suppressor gene (Cheng et al., 1994; Sekido et al., 1995). *BAP1*, encoding a nuclear deubiquitinase, was found to be mutated or deleted in multiple cancers, including ∼60% of human MM (Bott et al., 2011; Bueno et al., 2016; Carbone et al., 2012; Harbour et al., 2010; Hmeljak et al., 2018; Nasu et al., 2015; Testa et al., 2011). Furthermore, alterations in the Hippo pathway, mTOR, and chromatin modifiers were found in MM (Bueno et al., 2016).

The Polycomb Group (PcG) of proteins are chromatin modifiers essential for the maintenance of gene repression and stabilization of cell fates. They carry out their function via three major complexes: the Polycomb repressive complex 1 (PRC1), PRC2, and the Polycomb repressive deubiquitinase complex (Blackledge et al., 2015; Scheuermann et al., 2010; Sparmann and van Lohuizen, 2006). PRC2 can bind to chromatin and its catalytic unit EZH2 trimethylate H3K27, which is then recognized by PRC1, which monoubiquitinates H2A on K119 leading to chromatin compaction and gene repression (Bracken and Helin, 2009). Vice versa, variant PRC1 complexes can initiate H2A119ub1, which then, via adapter proteins, can recruit PRC2, causing H3K27 trimethylation (Blackledge et al., 2014). BAP1 opposes the function of PRC1 via the deubiquitination of H2A119ub1 as part of the Polycomb repressive deubiquitinase complex. Deregulation of the Polycomb repressive system is implicated in many cancers (Blackledge et al., 2015; Sparmann and van Lohuizen, 2006).

Several research groups have generated asbestos-induced mesothelioma in the mouse (Fleury-Feith et al., 2003; Kadariya et al., 2016; Marsella et al., 1997; Napolitano et al., 2016; Xu et al., 2014). Induction of mesothelioma in *Nf2*-deficient mice and *Cdkn2ab*-deficient mice and combined *Trp53-* and *Pten*-deleted mice (Altomare et al., 2011; Altomare et al., 2005a; Sementino et al., 2018) was reported. We previously reported that simultaneous inactivation of *Nf2* and *Trp53* in the mesothelial lining of the thoracic cavity of mice gives rise to mesothelioma. In addition, depleting *Cdkn2a* further accelerated tumor development (Jongsma et al., 2008).

Our compound mouse models harbor combinations of the predominant genetic lesions found in human MM. This results in a significantly faster onset of mesothelioma compared with current models that require a considerable time to develop MM. Besides, the models recapitulate well the cognate human disease, including the characteristic inflammatory microenvironment. Therefore, the models described here should be attractive for testing new therapeutic interventions.

## Results and discussion

### *Bap1* deletion together with *Nf2* and *Cdkn2ab* leads to a rapid onset of MM

**Figure 1.**
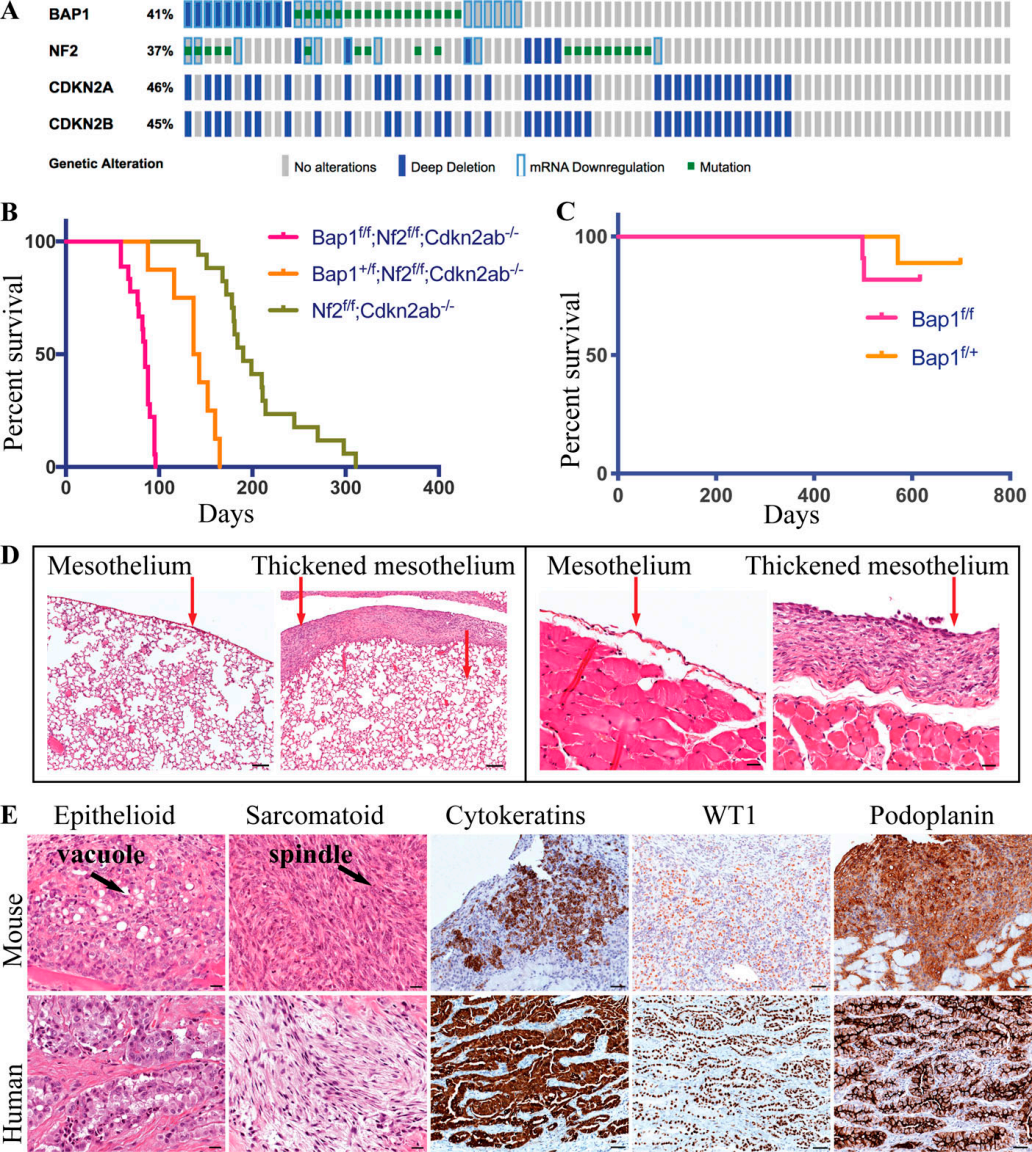
**Bap1 loss accelerates tumor initiation and progression in a mouse model of mesothelioma. (A)** CBioportal oncoplot of *BAP1*, *NF2*, *CDKN2A*, and *CDKN2B* in mesothelioma TCGA data. **(B)** Overall survival of Ad-CMV-Cre–activated *Bap1*^f/f^;*Nf2*^f/f^;*Cdkn2ab*^−/−^ (BNC; *n* = 18), *Bap1*^f/+^;*Nf2*^f/f^;*Cdkn2ab*^−/−^ (*n* = 8), and *Nf2*^f/f^;*Cdkn2ab*^−/−^ (NC; *n* = 17). **(C)** Survival curve of *Bap1*^f/f^ (*n *= 11) and *Bap1*^f/+^ (*n* = 9) mice. All mice have mixed C57BL/6 and FVB background. **(D)** Representative H&E staining of lungs and diaphragm of mouse at 4 wk after Adeno-Cre injection. Scale bars are 100 µm for the lung H&E and 20 µm for the diaphragm H&E. **(E)** H&E staining of human and mouse mesothelioma representing epithelioid and sarcomatoid cells. IHC of cytokeratins, WT-1, and podoplanin (D2–40) of both human and mouse mesothelioma. Scale bars are 20 µm for H&E and 50 µm for immunohistostaining.

**Figure S1.**
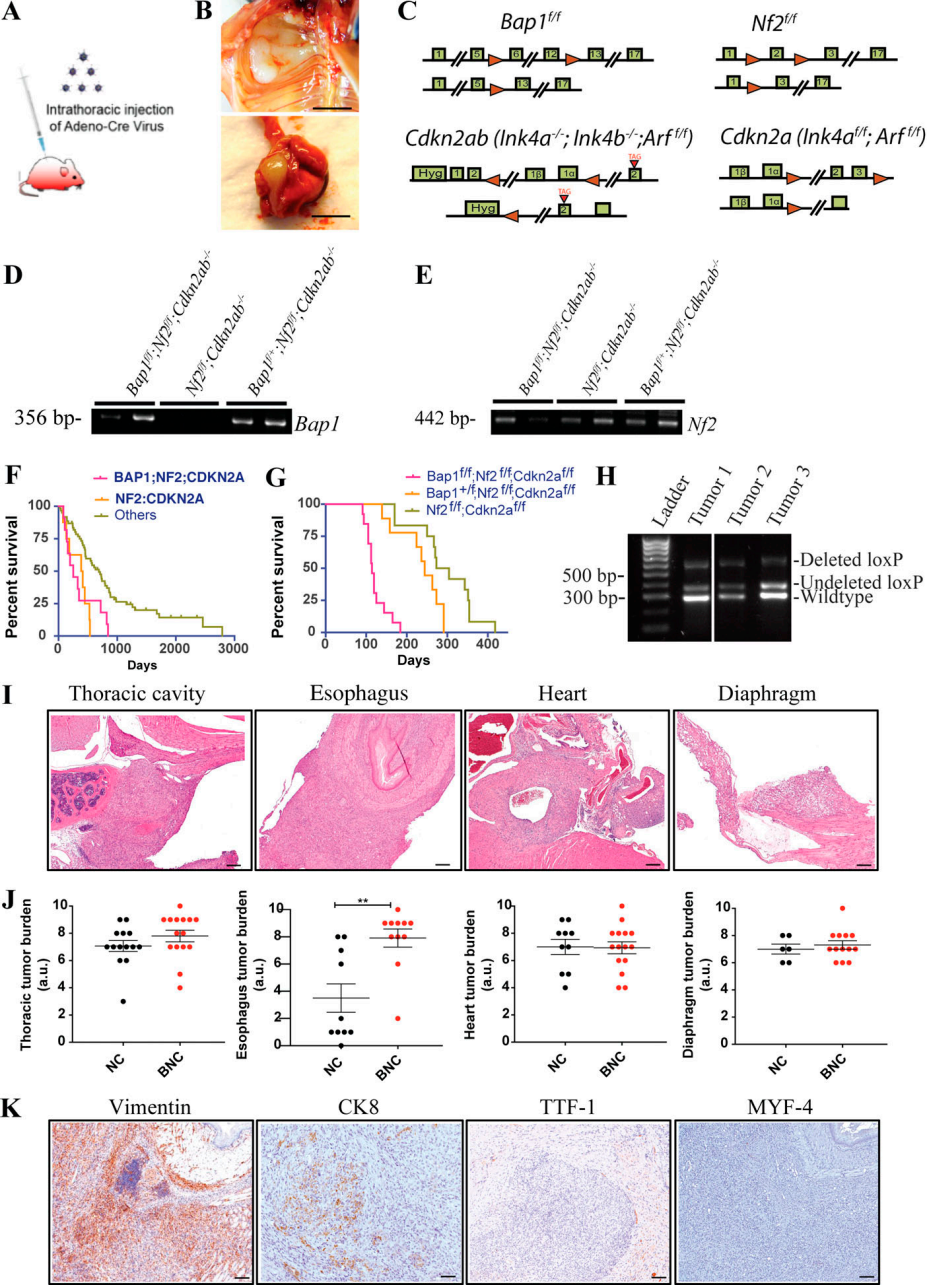
**BAP1 loss accelerates tumor development in a mouse model of mesothelioma. (A)** Schematic of intrathoracic injection of Adeno-Cre virus into the pleural space of a mouse by insulin injection syringe to induce deletion of floxed alleles in mesothelial layers. **(B)** Photographs of mesothelioma in thoracic wall and lungs/pleura. Scale bars are 5 mm. **(C)** Schematic of gene targeting in murine ES cells of the *Bap1* (obtained from the European Conditional Mouse Mutant repository consortium), *Nf2*, *Cdkn2a*, and *Cdkn2ab* loci. The *Cdkn2ab* mice are germline deleted for *Ink4a* and *Ink4b* but conditional for *p19Arf*. The *Cdkn2a* mice are conditional for *Ink4a* and *Arf*. The first row of each knockout strategy is the loci after loxp site (red triangles) insertion, and the second row represents loci after the Cre recombination. **(D)** PCR amplification of the flanking genomic DNA of the lox sites confirms the deletion of *Bap1* floxed alleles in mice MM tumors. **(E)** PCR confirms the deletion of *Nf2* floxed alleles in mice MM tumors. Presence of DNA band in gel indicates deletion of floxed alleles of *Bap1* and *Nf2*. **(F)** Overall survival of mesothelioma patients from the TCGA cohort with combined inactivation of *BAP1;NF2;CDKN2A*, *NF2:CDKN2A*, and other lesions. **(G)** Overall survival of Ad-CMV-Cre–activated *Bap1*^f/f^;*Nf2*^f/f^;*Cdkn2a*^f/f^ (BNC^a^; *n* = 13), *Bap1*^f/+^;*Nf2*^f/f^;*Cdkn2a*^f/f^ (*n* = 9), and *Nf2*^f/f^;*Cdkn2a*^f/f^ (NC^a^; *n* = 12). All mice are of mixed C57BL/6 and FVB background. **(H)** PCR amplification of wild-type band without any lox site confirms no LOH. **(I)** H&E image of mesothelioma of BNC mice in different organs of the thoracic cavity. Scale bars are 200 µm. **(J)** Quantification of tumor burden in different organs/location of BNC and NC mice with MM. The y axis represents values in the scale of 1 to 10. 10 is the highest tumor burden, and 0 is no tumor. The y axis values were assessed by the fraction of the tumor cells in histopathology slides of each organ. Unpaired Student’s *t* test was used to analyze the data in B. **, P < 0.01. Error bars represent mean ± SEM. **(K)** Representative IHC staining of vimentin, CK8, TTF-1, and MYF-4. Scale bars are 100 µm. For CK8, scale bar is 50 µm.

The Cancer Genome Atlas (TCGA) database shows that *BAP1* is frequently inactivated in conjunction with disruption of *NF2* and *CDKN2AB* (Fig. 1 A; Yap et al., 2017). To study the contribution of each of these genetic lesions to mesothelioma pathogenesis, we inactivated these genes in various combinations by injection of Adeno-Cre (Adenovirus carrying the Cre recombinase gene driven from the CMV promoter) into the pleural space of mice carrying conditional alleles of these genes (Fig. S1, A and C; Jongsma et al., 2008). The combined loss of *Bap1*, *Nf2*, *Cdkn2ab* (hereafter BNC) in the mesothelial lining of the thoracic cavity gave rise to mesothelioma in all mice of the cohort (Fig. 1 B and Fig. S1, B, D, and E). Mice in which *Nf2*, *Cdkn2ab* (hereafter NC) were deleted but with functional *Bap1* alleles gave rise to mesothelioma in 75% of the mice, whereas the remaining 25% succumbed from other tumors such as histiocytic sarcoma and lymphoma (Table S1). The mice were sacrificed when they showed signs of illness (respiratory distress, breathing abnormalities, and weight loss). The median survival of mice homozygously deleted for NC was 190 d. The additional heterozygous or homozygous loss of *Bap1* reduced the median survival to 140 and 85 d, respectively (Fig. 1 B). This is in line with the poor survival observed in man (Fig. S1 F), similar to analysis reported using the TCGA mesothelioma patient with *BAP1*, *NF2*, and *CDKN2A* alterations (Hmeljak et al., 2018).

The short latency of the BNC cohort required all mice to be sacrificed in a narrow time window between 8 and 14 wk after Adeno-Cre injection. Given the short latency period of the BNC cohort, tumor onset should be detectable at a much earlier time point. Therefore, we sacrificed mice at week 4 and found lesions that manifested as a thickened mesothelial lining covering the lung and thoracic walls, indicating swift and synchronous tumor development (Fig. 1 D). This implies that this combination of genetic lesions is sufficient to command rapid and reproducible MM development.

The tumors that arose in the BNC mouse model were mainly thoracic MM situated in the mediastinum, heart (mainly in the atrium), large blood vessels, esophagus, diaphragm, and thoracic wall (Fig. S1, B and I). The tumor burden in the esophagus of BNC mice is significantly higher than that seen in NC mice, whereas the tumor burden is similar in other organs of the thoracic cavity (Fig. S1 J). Microscopically, we observed multiple malignant neoplasias with different histological components ranging from epithelioid and sarcomatoid to biphasic. The thoracic MM in the BNC model is predominantly biphasic. Whether this feature of such lesions represents independently arisen epithelioid and sarcomatoid clones is unclear. The epithelioid cells showed nested, nodular, trabecular, and/or sheet-like arrangements, and the tumor cells were small to medium in size and round to oval in shape, and some contained rich foamy cytoplasm that often converted to large vacuoles (Fig. 1 E). The sarcomatoid cells had a short spindle-shaped appearance and were organized in fascicular structures (Fig. 1 E). Immunohistochemistry (IHC) for pan-cytokeratin, CK8, Wilms tumor-1 (WT-1), vimentin, and podoplanin confirmed the mesothelioma-specific characteristics of these lesions, which were similar to the human counterpart (Fig. 1 E and Fig. S1 K). Additionally, IHC staining of TTF-1 and MYF-4 was performed for differential diagnosis of lung adenocarcinoma and rhabdomyosarcoma, which can be misdiagnosed as MM in some cases. The negative staining for TTF-1 and MYF-4 indicates that the tumors do not represent adenocarcinoma or rhabdomyosarcoma and therefore are genuine MM (Fig. S1 K).

To understand the relative contribution of deletion of each of the *Bap1*, *Nf2*, *Cdkn2a*, and *Cdkn2b* alleles to mesothelioma development, we developed single and compound models with various combinations of inactivated alleles (Table S1). Deletion of *Bap1* alone in the thoracic cavity in a cohort of 20 mice did not result in mesothelioma during the lifetime of the mice (monitored for up to 700 d) except in one heterozygous floxed mouse (Fig. 1 C and Table S1). Previously, we reported that genetic deletion of *Nf2* or *Cdkn2a* alone does not induce mesothelioma (Jongsma et al., 2008; Krimpenfort et al., 2007). The compound models with deletion of *Bap1*, *Nf2,* and *Cdkn2a* (*Ink4a/Arf* conditional; hereafter BNC^a^) and *Nf2* together with *Cdkn2a* (hereafter NC^a^) gives rise to mesothelioma in mice with median survival of 114 and 244 d, respectively (Fig. S1 G). This BNC^a^ and NC^a^ cohort showed MM with epithelioid, biphasic, and sarcomatoid histological subtypes while a similar study reported only biphasic and sarcomatoid MM (Kukuyan et al., 2019). Heterozygous deletion of *Bap1* in either NC or NC^a^ mice showed significant tumor acceleration without evidence of loss of heterozygosity (LOH) in the tumors, indicative of a dose-dependent effect of *Bap1* on tumor development (Fig. S1 H). Collectively, these mouse models show a strong MM accelerating effect of *Bap1* loss in conjunction with the deletion of *Nf2* and *Cdkn2a/Cdkn2ab*.

### BAP1-deficient mesothelioma shows augmented activation of PI3K and MAPK pathways

**Figure 2.**
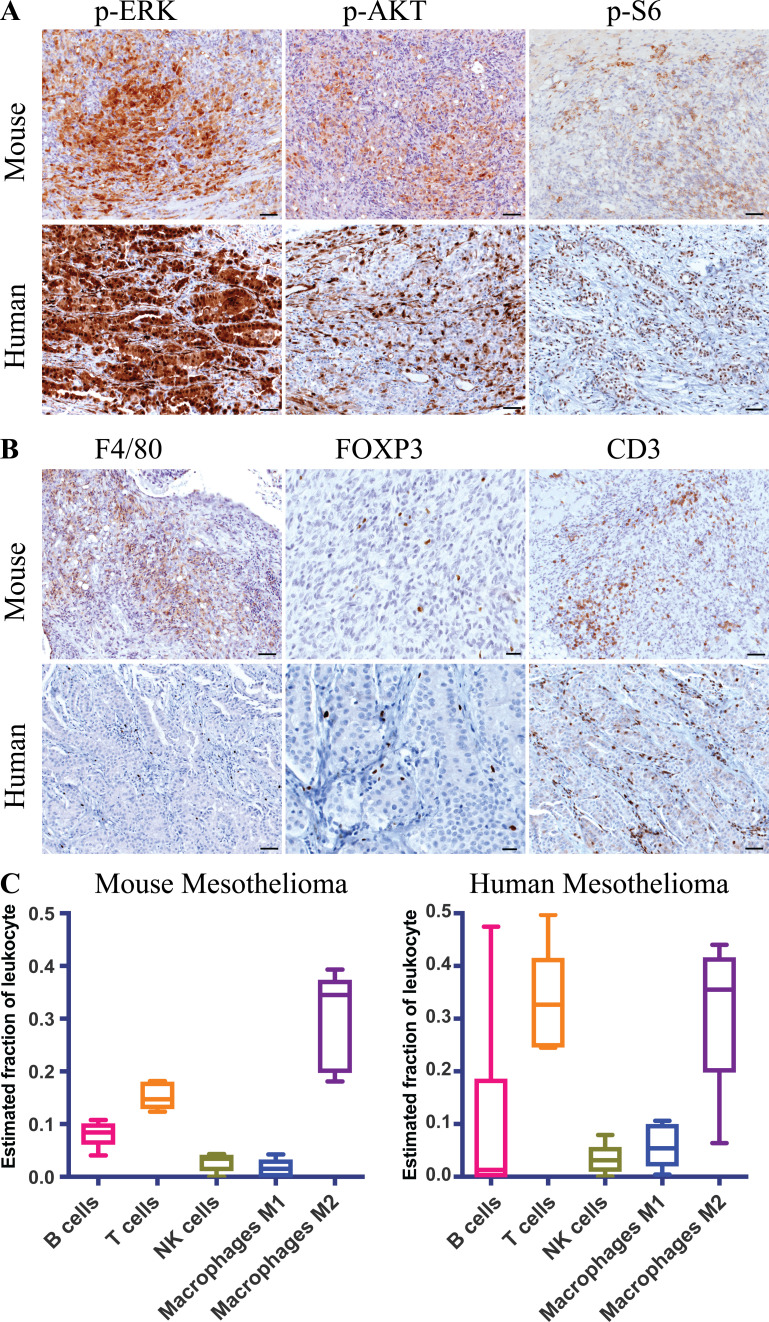
**Mouse BNC mesothelioma shows hyperactivation of MAPK and PI3K pathways and inflammatory tumor microenvironment. (A)** IHC staining of p-ERK, p-AKT, and p-S6 of both human and mouse mesothelioma. **(B)** IHC staining of F4/80 or CD68, FOXP3, and CD3 of both human and mouse mesothelioma. Scale bars are 50 µm in A and B, except for FOXP3, which is 20 µm. **(C)** Box and whisker plot of the estimated immune cell composition in fraction of BNC mouse tumors (*n* = 5) and human mesothelioma tumors (*n* = 6) as determined by CIBERSORT. The line inside the box is median. The bottom and the top of the box are the lower and upper quartile respectively. Error bars on the whiskers represent min to max.

**Figure S2.**
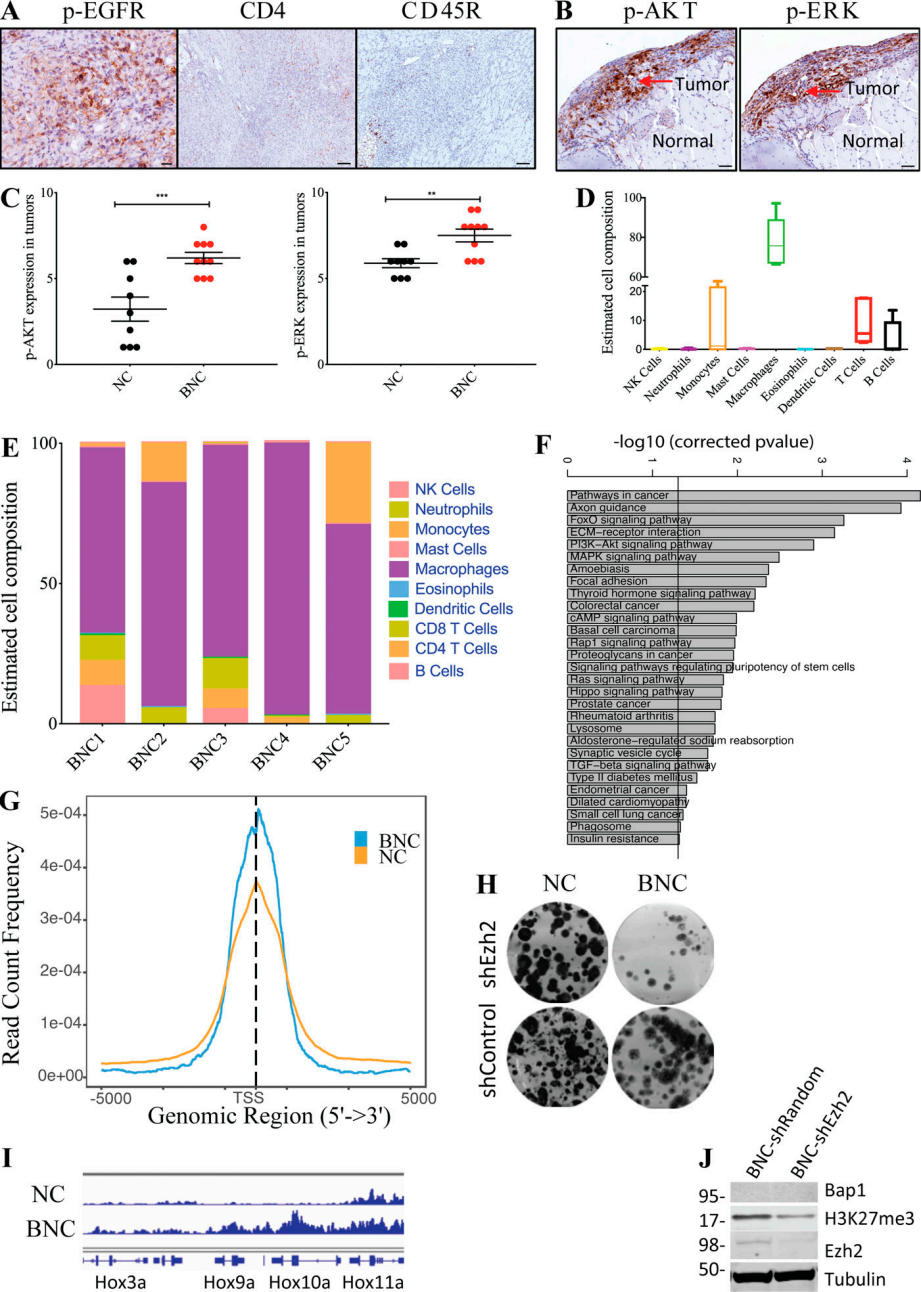
**Mouse BNC mesothelioma shows hyperactivation of MAPK and PI3K pathways and exhibits an inflammatory tumor microenvironment. (A)** Representative IHC staining of p-EGFR, CD4, and CD45R of mouse mesothelioma. Scale bar is 20 µm for p-EGFR and 50 µm for CD4 and CD45R. **(B)** IHC staining of p-ERK, and p-AKT of mouse mesothelioma at 4 wk after Adeno-Cre injection. Scale bars are 50 µm. **(C)** Quantification of p-AKT and p-ERK staining in BNC (*n* = 10) and NC (*n* = 9) mice with MM. The y axis represents values in the scale of 1 to 10. 10 is the highest (staining intensity + fraction of tumor cells) burden, and 0 is no staining. Unpaired Student’s *t* test was used to analyze the data in B. **, P < 0.01, ***, P < 0.001. Error bars represent mean ± SEM. **(D)** Box plot of estimated cell composition of 10 immune cell types in percentage in five independent BNC mouse mesothelioma tumors by Seq-ImmuCC. Error bars on the whiskers represent min to max. **(E)** Bar plot showing relative immune cell estimates of 10 different immune cells in five BNC tumors as determined by Seq-ImmuCC. **(F)** Plot showing significantly enriched KEGG pathways in overexpressed genes in BNC mesothelioma cells. **(G)** Plot showing enrichment of H2A119ub1 binding in TSS in both NC and BNC mesothelioma cells. **(H) **Clonogenicity assay of BNC and NC mesothelioma cells upon EZH2 inhibition by shRNA. **(I)** Enrichment plot showing normalized binding in the Hox cluster of both BNC and NC mesothelioma cells. Data are based on at least two independent experiments. **(J)** Immunoblot showing BAP1, H3K27me3, EZH2, and Tubulin upon *Ezh2* knock-down by shRNA in BNC and NC cells.

In mesothelioma, multiple oncogenic pathways have been implicated, including the PI3K, MAPK, and Hippo pathways (Altomare et al., 2005b; Marek et al., 2014; Menges et al., 2010; Miyanaga et al., 2015; Ramos-Nino et al., 2005; Shukla et al., 2011; Suzuki et al., 2009). We observed prominent MAPK pathway activation as evidenced by phosphorylated ERK (p-ERK) and phosphorylated epidermal growth factor receptors (p-EGFR) staining in both BNC and NC mice (Fig. 2 A and Fig. S2, A and B). PI3K pathway activation was demonstrated by p-AKT expression in the tumor specimen, with patchy and/or clustered staining patterns reflecting tumor heterogeneity in both human and mouse MM (Fig. 2 A). The downstream effectors of these pathways, such as p-S6 kinase, also stain positively in both human and mouse mesothelioma (Fig. 2 A). The p-AKT and p-ERK levels are significantly higher in BNC tumors than NC tumors (Fig. S2 C). The enhanced expression of genes acting in the PI3K, MAPK, and Hippo pathways in *Bap1*-deficient mouse mesothelioma cells is in line with these findings (Table S1). In summary, our mouse model closely recapitulates the activation of the signaling pathways observed in human mesothelioma with BAP1 loss, which acts as a potent tumor accelerator in mice and therefore likely also in humans.

### BNC-derived tumors recapitulate the immunophenotype of human mesothelioma

Inflammation is closely associated with human MM and is ascribed to a response inflicted by exposure to asbestos fibers (Robinson and Lake, 2005; Shrestha et al., 2019). Interestingly, our BNC mesothelioma model, which is exclusively based on engineered tumor suppressor gene deletions, closely mimics this inflammatory phenotype. We observed substantial macrophage infiltration as shown by IHC staining of F4/80 and CD68 (Fig. 2 B). As is the case for human mesothelioma, we noted significant numbers of T cells, including regulatory T cells as shown by CD3, CD4, and FOXP3 staining, B cells, and natural killer (NK) cells (marked by CD45R) in BNC tumors (Fig. 2 B and Fig. S2 A). Notably, ingenuity pathways analysis (IPA) of the differentially expressed genes in BNC tumors compared with NC tumors shows enrichment of NF-κB neuroinflammatory signaling, a known mediator of inflammation that likely contributes to the recruitment of immune cells to these tumors (Table S1; Liu et al., 2017). We also assessed the immune cell composition by Seq-ImmuCC in BNC MM (Chen et al., 2018). This analysis confirmed the widespread presence of immune cells, including T cells and macrophages, in BNC tumors (Fig. S2, D and E). Subsequently, we made an estimate of the immune cell subtypes by CIBERSORT in BNC tumors and compared these to asbestos-exposed human mesotheliomas carrying combined *BAP1*, *NF2*, and *CDKN2A* alterations (Newman et al., 2015). This analysis indicated that M2 macrophages, T cells, and B cells comprise a major fraction of the leukocyte population in both BNC and human mesothelioma with BAP1, NF2, and CDKN2A loss (Fig. 2 C). This is relevant, as the immunophenotype observed in man is often ascribed to asbestos exposure. However, our results show that even without asbestos exposure and associated DNA damage, the combined deletion of the tumor suppressors *Bap1*, *Nf2*, and *Cdkn2ab* creates a mesothelioma-specific microenvironment that enables a similar immunophenotype (Fig. 2 C). The immunophenotype observed in BNC mice is similar to that in NC mice and therefore is not unique to *Bap1* loss. This indicates that the tumor subtype itself plays a major role in dictating the immune-related characteristics of the tumor microenvironment.

### BNC mouse MM cells are sensitized to PI3K inhibition, radiation, and PARP inhibition

**Figure 3.**
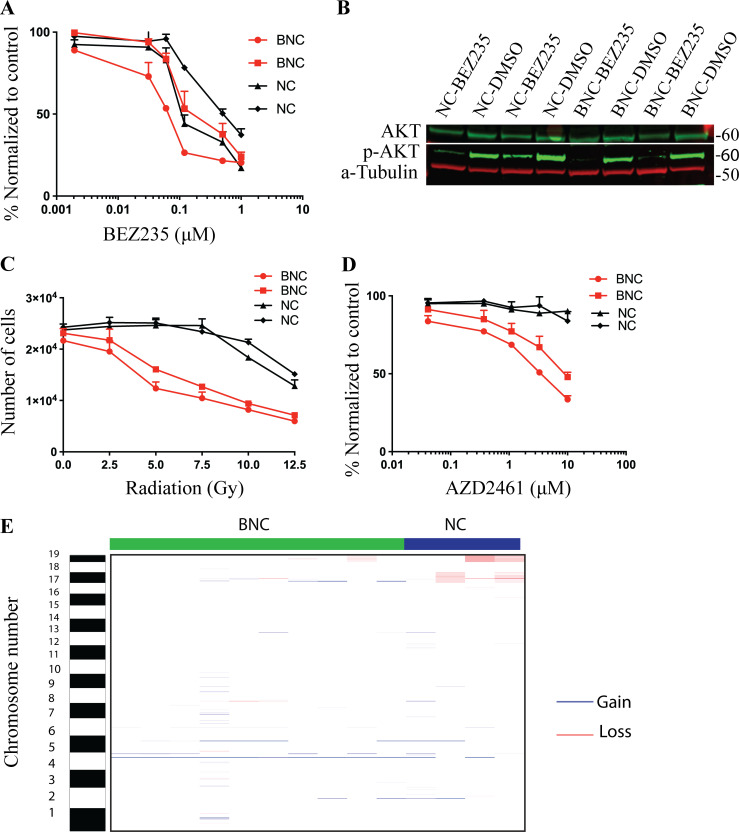
**Mouse and human mesothelioma cells hypersensitive to PI3K inhibition, PARP inhibition, and radiation. (A)** Dose-response curve of BNC (*n* = 2) and NC (*n* = 2) mesothelioma cells to PI3K inhibitor BEZ-235. **(B)** Immunoblot showing p-AKT, total AKT, and Tubulin in BEZ-235–treated BNC (*n* = 2) and NC (*n* = 2) mesothelioma cells. **(C and D)** Dose-response curve of two independent BNC and NC mesothelioma cells to γ-radiation (C) and PARP inhibition (D). **(E)** Copy number alteration profile of genomic losses and gains as determined by CGHcall in both BNC (*n* = 10) and NC (*n* = 4) tumors.

To determine whether BAP1 depletion confers a distinct drug response profile, we assessed the response of BAP1-deficient and -proficient cell lines to PI3K inhibition, γ-radiation, and poly(ADP-ribose) ploymerase (PARP) inhibition. The mouse cells used here were early passage cell lines (within the first four to eight passages) derived from BNC and NC tumors. Mouse MM cells are sensitive to the PI3K inhibitor BEZ-235 as observed in human mesothelioma (Fig. 3 A; Altomare et al., 2005b). We observed a significant impaired phosphorylation of AKT by BEZ-235 (Fig. 3 B). This indicates that the hyperactivation of the PI3K pathway sensitizes mesothelioma to PI3K inhibition irrespective of BAP1 status (Fig. 3 B). The literature suggests that BAP1 promotes DNA double-strand break repair and that its loss leads to homologous recombination repair defects, reminiscent of BRCA1/2 deficiency. Therefore, we tested the sensitivity of BAP1-proficient and -deficient cells to radiation and PARP inhibition. *Bap1 *deletion causes increased sensitivity of mesothelioma cells to γ-radiation and PARP inhibition, a feature seen in multiple human BAP1-deficient cell types (Peña-Llopis et al., 2012; Yu et al., 2014; Fig. 3, C and D). We reasoned that loss of BAP1 may contribute to the extent of copy number alterations seen in mesothelioma given its role in DNA damage repair. However, we did not observe major recurrent chromosome segment losses or gains in either BNC or NC tumors with the exception of chromosomes 17 and 19 (Fig. 3 E). This indicates that BAP1 loss did not significantly augment genomic instability in our BNC mouse model. The lack of major genomic changes in BNC points toward other mechanisms of PARP inhibitor sensitivity.

### The BNC autochthonous model allows for fast preclinical testing of treatment modalities

**Figure 4.**
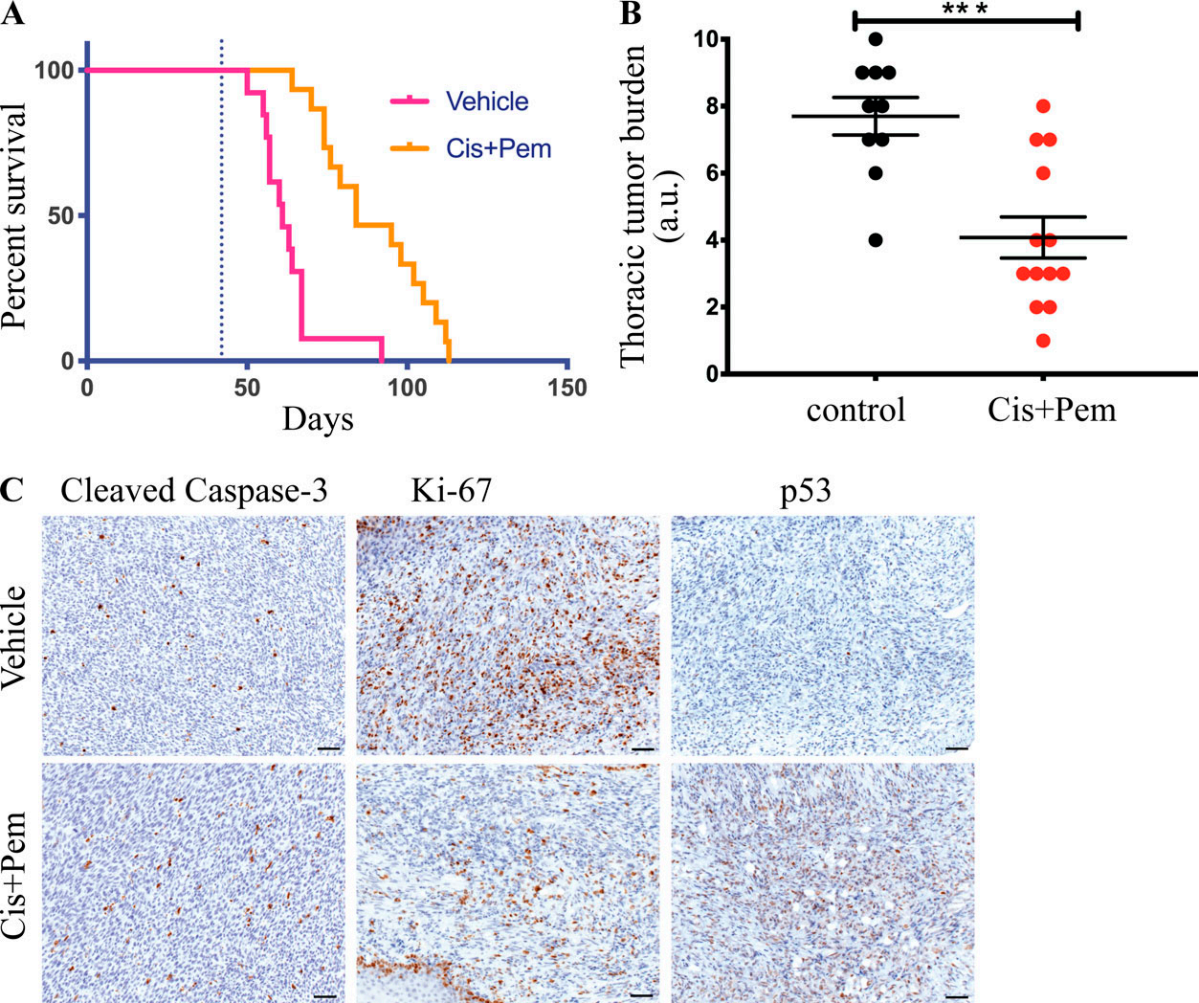
**Mouse BNC mesothelioma shows improved survival upon cisplatin plus pemetrexed treatment. (A)** Survival curve of vehicle- (*n* = 13) and cisplatin + pemetrexed (Cis+Pem)–treated (*n* = 15) BNC mouse. **(B)** Quantification of tumor burden in the thoracic cavity in treated and control cohorts. The y axis represents values in the scale of 1 to 10. 10 is the highest tumor burden in that cohort, and 0 is no tumor. Error bars represent mean ± SEM. **(C)** Cleaved Caspase-3, Ki-67, and p53 staining of treated and untreated BNC tumors. Scale bars are 20 µm. All mice used had mixed C57BL/6 and FVB background. Littermates were used in this experiment. Unpaired Student’s *t* test was used to analyze data in B. ***, P < 0.001.

In the clinic, the frontline treatment approved for mesothelioma is cisplatin in combination with either pemetrexed or raltitrexed. This yields a modest survival benefit (Ladanyi et al., 2012; Vogelzang et al., 2003). We tested how BNC mice respond to this first-line standard treatment. We started administration of cisplatin and pemetrexed 6 wk after deletion of BNC alleles. We followed the mice until they showed signs of respiratory distress and significant weight loss (considered the endpoint). This treatment prolonged survival of the mice by approximately 3 wk, with an increased median survival of treated mice to 84 d compared with 61 d for untreated mice (Fig. 4 A). Additionally, the thoracic tumor burden of the cisplatin and pemetrexed–treated mice appeared significantly less than that of the vehicle control mice (Fig. 4 B). Treated tumors exhibited a significant increase in caspase-3 cleavage concomitant with a significantly impaired proliferation, as shown by Ki-67 staining (Fig. 4 C). Taken together, the limited survival advantage conferred by this drug combination mimics the treatment response and chemotherapy resistance seen in human MM patients. We noted a substantial induction of p53 protein under these circumstances (Fig. 4 C). Therefore, further potentiating the apoptosis-inducing arm of p53 could be an attractive treatment strategy in MM.

### *Bap1* loss results in a largely repressive transcriptional program

**Figure 5.**
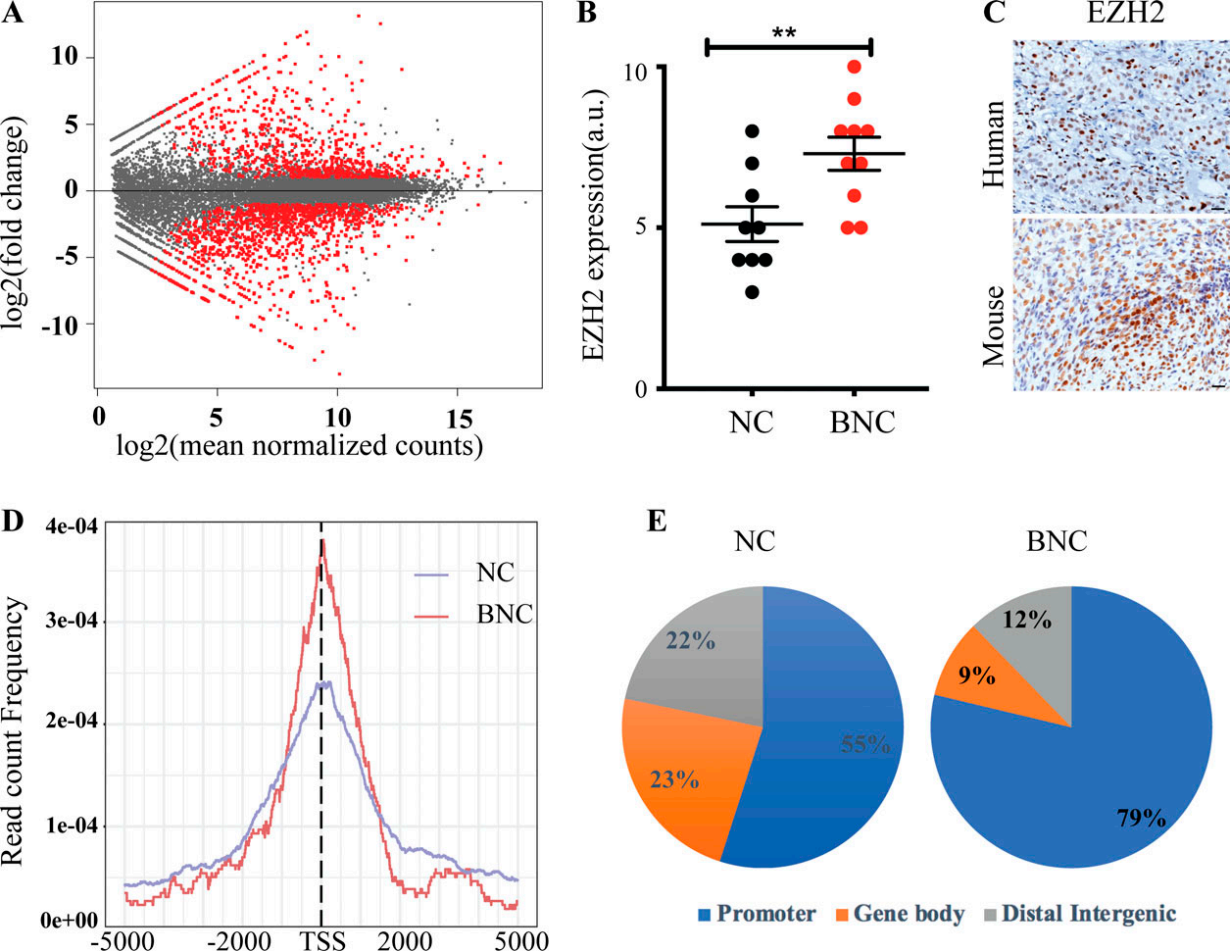
**BAP1 loss drives a largely repressive transcriptional program. (A)** Red dots in MA plot showing differentially expressed genes of BNC (*n* = 3) versus NC (*n* = 3) mesothelioma cells (P < 0.01 is significant; P value adjusted for multiple testing using Benjamini-Hochberg). **(B)** Quantification of EZH2 expression in NC (*n* = 8) and BNC (*n* = 10) MM tumors. 10 is the highest expression of EZH2, and 0 is no expression on the y axis. Error bars represent mean ± SEM. **(C)** Representative of IHC staining of EZH2 of both human and mouse BNC mesothelioma. Scale bars are 20 µm. **(D)** Plot showing enrichment of H3K27me3 binding in TSS in both BNC and NC mesothelioma cells. **(E)** Pie chart showing distribution of H3K27me3 binding in both BNC and NC mesothelioma cells. Data are based on at least two independent experiments. Promoter represents ±5 kb of TSS. Unpaired Student’s *t* test was used to analyze data in B. **, P < 0.01.

To understand the molecular consequences of BAP1 depletion, we determined the gene expression profile of primary tumor cell lines by RNA sequencing (RNA-seq). We performed differential mRNA expression analysis between BNC- (*n* = 3) and NC-depleted (*n* = 3) lines. We found 1,954 deregulated genes (P < 0.01 and greater than twofold up-regulated or down-regulated), of which 1,182 were down-regulated and 772 were up-regulated (Table S1). A large fraction of the genes were down-regulated in *Bap1*-deficient compared with *Bap1* wild-type mouse mesothelioma cells (Fig. 5 A). This is in line with the TCGA human mesothelioma data in which 75% of the genes with altered expression in *BAP1*-deficient samples compared with *BAP1* wild-type samples are down-regulated (Hmeljak et al., 2018). Additionally, Kyoto Encyclopedia of Genes and Genomes (KEGG) pathway enrichment of genes deregulated in BNC mesotheliomas revealed an overall prevalence of genes implicated in PI3K, MAPK, FAK, and Hippo signaling among others (Fig. S2 F).

Loss of tumor suppressor BAP1 causes EZH2-dependent myeloid transformation and promotes tumorigenesis in cells that do not engage an RNF2-dependent apoptotic program (Dey et al., 2012; He et al., 2019; LaFave et al., 2015). Epigenetic modulation, in particular by EZH2, may play a critical role in MM with BAP1 loss. Indeed, we observed that loss of BAP1 results in elevated EZH2 expression and increased H3K27me3 levels, and knock-down of *Ezh2* leads to decreased H3K27me3 levels in BNC cells (Fig. 5, B and C; and Fig. S2 J). To gain further insight into how *Bap1* loss–induced EZH2 up-regulation may influence H3K27 methylation and promote faster tumor development, we exploited genome-wide chromatin profiling in mouse mesothelioma–derived primary tumor cells. Identification of several evolutionary conserved PRC2 targets such as *Hox* genes underlined the reliability of the chromatin profiling (Fig. S2 I). The global promoter site (±5 kb of transcription start site [TSS]) occupancy of H3K27me3 in BAP1-deficient cells (78%) is significantly higher than in BAP1-proficient cells (55%; Fig. 5, D and E). This shift to promoter-centric binding was also observed for the histone mark H2A119ub1 in BAP1-deficient cells (Fig. S2 G). This implies a redistribution of repressive marks in BAP1-depleted cells toward promoter sites, leading to global down-regulation of multiple genes as seen in both mouse cell lines and human TCGA data (Fig. 5 A; Hmeljak et al., 2018; LaFave et al., 2015). In line with the observations in myeloid cells, *Bap1*-deleted mouse MM cell proliferation is impaired upon shRNA-mediated down-regulation of *Ezh2* (Fig. S2 H; LaFave et al., 2015). Collectively, our data point to a situation in which BAP1 depletion in mesothelioma promotes polycomb-mediated gene repression, a tumor-promoting event seen in many tumor settings.

In conclusion, we have described here a series of autochthonous mouse models for mesothelioma based on the most frequently occurring mutations in human MM. Among them, tumors in the BNC model develop as fast as a cell line–based tumor graft model. Furthermore, it exhibits an inflammatory immunophenotype reminiscent of human mesothelioma. The model is relatively “clean” and unlikely to suffer from the accumulation of many additional lesions. This offers the possibility of investigating the underlying tumor suppressor pathways that are connected to increased Polycomb repression upon loss of *Bap1*. Given the extremely fast tumor development, the model is particularly suitable for testing new treatment modalities. In addition, the model provides the opportunity to investigate chemotherapy resistance mechanisms as well as to conduct screens for new synthetic lethal drug combinations. Finally, being immunocompetent, the BNC model is a valuable addition to immunodeficient mesothelioma models based on patient-derived xenografts, permitting exploration and validation of new immunotherapy concepts in MM.

## Materials and methods

### Conditional knockout generation and genotyping

*Bap1* conditional mice were generated using embryonic stem (ES) cells with conditionally targeted exons 6–12 of *Bap1* gene with *loxP* sites. These ES cell clones were obtained from the European Conditional Mouse Mutant repository. The *Nf2* conditional deletion mouse has been described by Giovannini et al. (2000), and Cdkn2a and *Cdkn2ab* mice have been described by Krimpenfort et al. (2007). The conditional knockout alleles for *Bap1*, *Nf2*,* Cdkn2a*, and *Cdkn2ab* are schematically depicted in Fig. S1 C. Southern blot–verified ES cells were expanded and injected into a blastocyst to generate chimeric mice. Chimeras were then bred with BL/6 mice to obtain germline-transmitting *Bap1^frt;f/+^* mice. Subsequently, these mice were crossed with Flpe mice to remove the lacZ cassette from the *Bap1* locus. *Bap1*^f/f^, *Bap1*^f/+^, and *Bap1^+/+^* littermate mice were genotyped by PCR with the primers Bap1F1 (5′-CTC​AAT​ATT​CCA​CCC​TGC​GTC​TG-3′), Bap1R1 (5′-GGC​AGG​TGG​CCT​CCT​CTA​CTC​TA-3′) using the following parameters: 95°C for 5 min, followed by 30 cycles of 94°C for 30 s, 56°C for 30 s, and 72°C for 40 s, and then 72°C for 5 min. The wild-type allele was detected at 250 bp, and the floxed allele was detected at 356 bp by PCR. The deletion of the genomic region of *Bap1* and *Nf2* flanked by Lox sites was determined by PCR amplification using the following primers: Bap1_Up: 5′-ACT​GCA​GCA​ATG​TGG​ATC​TG-3′, Bap1R1: 5′-GGC​AGG​TGG​CCT​CCT​CTA​CTC​TA-3′, Nf2_P5: 5′-GAA​GGC​AGC​TTC​CTT​AAG​TC-3′, and Nf2_P6: 5′-CTC​TAT​TTG​AGT​GCC​TGC​CAT​G-3′. To assess the LOH of the wild-type *Bap1* allele in *Bap1^f/+^;Nf2^f/f^;Cdkn2ab^−/−^* tumors, we used the following three primers for PCR amplification: Bap1_Up: 5′-ACT​GCA​GCA​ATG​TGG​ATC​TG-3′, Bap1R1: 5′-GGC​AGG​TGG​CCT​CCT​CTA​CTC​TA-3′, and Bap1F1: 5′-CTC​AAT​ATT​CCA​CCC​TGC​GTC​TG-3′. The lower band represents the undeleted genomic region of wild-type *Bap1 *(Fig. S1 H). The PCR reactions were performed using the following parameters: 95°C for 5 min, followed by 30 cycles of 94°C for 30 s, 56°C for 30 s, and 72°C for 40 s, and then 72°C for 5 min for *Bap1* deletion and LOH. PCR was performed using the following parameters: 95°C for 5 min, followed by 30 cycles of 94°C for 30 s, 58°C for 30 s, and 72°C for 40 s, and then 72°C for 5 min for *Nf2* deletion.

All animal work was performed according to protocols approved by institutional committees overseeing animal experiments (Animal experiment committee and Animal welfare committee) of The Netherlands Cancer Institute, Amsterdam, Netherlands. Mice were housed under standard feeding, light, and temperature with ad libitum access to food and water. All animals used had a mixed genetic background (FVB, Bl6, 129Ola).

### Deriving and propagating mesothelioma cell lines

A small piece of mesothelioma primary tumor was chopped into fine pieces and put in medium (DMEM/F12 [1:1]; GIBCO), supplemented with Glutamax, 4 µg/ml hydrocortisone (Sigma), 5 ng/ml murine EGF (Invitrogen), insulin-transferrin-selenium solution (GIBCO), 10% FCS (GIBCO), and penicillin and streptomycin (GIBCO). The chopped tissues were grown in 37°C in 5% CO_2_ for 2–3 d. The cells that attached to the culture dish and grew out were passaged, and cell lines were established.

### Induction of mesotheliomas in mice

6–8-wk-old mice were treated with 0.1% cyclosporine A (Novartis) in drinking water 1 wk before the adenovirus administration and 2–3 wk following the infection (Sutherland et al., 2011). The mice were injected intrathoracically with 10^9^ PFU purified adenovirus carrying Cre recombinase driven from the ubiquitous CMV promoter (Meuwissen et al., 2001). For injection, the mice were temporarily sedated with ketamine:sedazine:NaCl (2:1:17) by injecting 10 µl per gram of mouse weight intraperitoneally. The injection site was cleaned with 70% alcohol, and 50 µl virus particles was injected with an insulin injection needle in between the ribs into the pleural space (needle penetrated chest wall from 2 to 3 mm), and the contents of the syringe were slowly released (Fig. S1 A).

### Endpoint of the experiment and pathology of tumors

We put the mice on tumor watch after deletion of floxed alleles by Adeno-CMV-Cre. The mice were monitored daily for weight loss and breathing difficulties. We sacrificed the mice when they showed signs of illness (breathing abnormalities, hunch back, and weight loss) as the endpoint. For histological analysis, lungs were inflated with formalin or ethanol acetic acid/formalin. Other tissue-harboring tumors were collected separately and also fixed with formalin or ethanol-acetic acid/formalin for 24–48 h. Fixed tissues were subsequently dehydrated and embedded in paraffin, and 2-µm sections were cut and stained for H&E. For IHC, tissue sections were rehydrated, blocked in BSA containing PBS, and subsequently incubated with primary antibodies and subsequently with secondary antibodies. IHC was performed for proteins such as WT-1, cytokeratins, CK8, vimentin, F4/80, TTF-1, MYF4, CD68, CD3, CD4, CD45R, and FOXP3. Signaling pathways were examined by IHC with the following antibodies: p-EGFR, p-S6, p-AKT, and p-ERK. Sources of the antibodies are provided in Table S1.

### Patient selection

This work was done under the research protocol approved by the institutional review board of the Netherlands Cancer Institute. All patients whose materials were used provided written informed consent for the use and storage of tumor biopsies and germline DNA. We selected 12 patients based on the histological subtype, i.e., epithelioid (*n* = 7) and biphasic (*n* = 5), diagnosed by an expert human pathologist. Of these 12 tumors, 6 tumors were BAP1 negative as determined by the loss of nuclear staining in IHC staining. Tumor tissue sections of 2 µm were stained with H&E. IHC was performed with the following antibodies: p-S6, p-AKT, p-ERK, CD68, CD3, FOXP3, cytokeratins, WT1, and podoplanin.

### Knock-down of *Ezh2* and *Bap1* by shRNA

To knock down *Ezh2* expression in mesothelioma cells, we used dox-inducible FH1-tUTG-RNAi vectors containing the following targeting sequence: Ezh2-tetKD-A, 5′-GCA​AAG​CTT​GCA​TTC​ATT​TCA-3′ (Herold et al., 2008). As a control, we used Random-tetKD containing the targeting sequence 5′-ATT​CTT​ACG​AAA​CCC​TTA​G-3′. To knock down *Bap1*, we used shRNA constructs against mouse *Bap1* in the pLKO.1-puro vector from Sigma (*shBap1*_1: TRCN0000030719, *shBap1*_2: TRCN0000030723). Lentivirus was generated using a third-generation, tat-free packaging system.

### Immunoblotting

Whole cell extracts were prepared in radioimmunoprecipitation assay buffer (50 mM Tris, pH 8.0; 50 mM NaCl; 1% NP-40; 0.5% sodium deoxycholate; and 0.1% SDS) containing a protease inhibitor cocktail (Roche; Complete #11873580001). Equal amounts of protein as determined by BIO-RAD Protein Assay kit (#500–00006) were resolved on NuPage-Novex, 4%–12% Bis-Tris gels (Life Technology; #NP0322BOX) and transferred onto nitrocellulose membrane (GE Healthcare; Amersham Protran 0.2 µm NC, #10600001) using Thermo Fisher Xcell apparatus according to the manufacturer’s protocol. After incubation with 5% BSA dissolved in PBS containing 0.1% Tween 20 (PBST) for 2 h, the membrane was incubated with primary antibody against BAP1 (CST; #13271), EZH2 (BD Bioscience; #612666), H3K27me3 (CST; #9733), total AKT (#SC-8312), pAKT (CST; #4060), and α-tubulin (Sigma; T9062) at 4°C for 12–16 h. Membranes were washed three times for 15 min and incubated with HRP conjugated anti-mouse or anti-rabbit antibodies for 2 h. Blots were washed with PBST three times for 15 min and developed with the ECL system and scanned with ChemiDoc (BIO-RAD). Additionally, protein levels were measured in either the 700- or 800-nm channel using the Odyssey Infrared Imaging system (LI-COR) after incubation with appropriate secondary antibodies labeled with IRDye680 or IRDye800 fluorescent dyes (LI-COR), respectively.

### RNA isolation, gene expression analysis

RNA was isolated from tumor cell lines and mouse tumors with the Qiagen All Prep DNA/RNA kit. Quantification and quality assessments for RNA were performed with a Bioanalyzer (RNA integrity number >6.5; Agilent). Sequencing libraries were constructed with a TruSeq mRNA Library Preparation Kit using poly-A–enriched RNA (Illumina). The samples were run on a HiSeq 2500 Illumina sequencer generating 65-bp single-end reads. Raw sequence data and read counts data have been deposited in the Gene Expression Omnibus database (accession no. GSE145022). The sequence reads were mapped to the mouse genome (mm10) using TopHat (2.0.12). TopHat was run with default. Reads with mapping quality <10 and nonprimary alignments were discarded. Remaining reads were counted using HTSeq-count. Statistical analysis of the differential expression of genes was performed using DESeq2 (Love et al., 2014). Genes with false discovery rate for differential expression <0.01 were considered significant.

### Genomic DNA isolation, low coverage copy number sequencing

Genomic DNA was isolated from frozen tumor samples using the Allprep DNA/RNA/Protein mini kit (Qiagen) according to the manufacturer’s instructions. The amount of double-stranded DNA in genomic DNA samples was quantified using the Qubit dsDNA HS Assay Kit (Invitrogen). Subsequently, 250 ng of double-stranded genomic DNA was fragmented by Covaris shearing, and samples were purified with the Agencourt AMPure XP PCR Purification Beads according to the manufacturer's instructions (Beckman Coulter; #A63881). DNA library preparation for Illumina sequencing was done with the TruSeq DNA LT Sample Preparation kit (Illumina). Up to 10 uniquely indexed samples were pooled equimolarly, and each pool was then sequenced as single-end 65-bp run using an Illumina HiSeq2500 machine according to the manufacturer’s instructions.

Reads were aligned to the reference genome (mm10) using the Burrows-Wheeler Aligner (BWA 7.10). The CopywriteR program was adapted for low-coverage sequencing without peak calling algorithm (Kuilman et al., 2015). A depth-of-coverage method was used for 20-kb bins, and the read count was normalized for GC content and mappability. Log_2_-transformed ratios were calculated for all tumor samples versus reference (normal tail) samples. The normalized and corrected profiles were further analyzed by CGHcall (van de Wiel et al., 2007). Raw sequence data and log_2_ read count data have been deposited in the Gene Expression Omnibus database (accession no. GSE145022).

### Seq-ImmuCC and IPA

We did run the Seq-ImmuCC program on a web server to generate a comprehensive signature of immune cell composition in mouse tumor as described (Chen et al., 2018). It provides a quantification of 10 different immune cell types from the RNA-seq data of mouse tissues. For pathways analysis, we used the IPA core program. Selected molecular and cellular functions predicted to be enriched by the differentially expressed genes were ranked in order of significance.

### Enumeration of immune cell types using mRNA expression data

CIBERSORT software was applied to RNA-seq gene expression data to estimate the proportions of 22 immune cell types (B cells naive, B cells memory, plasma cells, CD8 T cells, naive CD4 T cells, resting memory CD4 T cells, activated memory CD4 T cells, follicular helper T cells, gamma delta T cells, regulatory T cells [T reg cells], resting NK cells, activated NK cells, monocytes, M0 macrophages, M1 macrophages, M2 macrophages, resting dendritic cells, activated dendritic cells, resting mast cells, activated mast cells, eosinophils, and neutrophils) using the LM22 dataset provided by the CIBERSORT platform. We selected patients from TCGA mesothelioma cohorts with combined BAP1, NF2, and CDKN2A alterations and history of asbestos exposure. The RNA-seq data of the selected patients and BNC mouse tumors were used for CIBERSORT analysis. Before running the CIBERSORT, we changed the mouse gene names to the corresponding human orthologue gene names, as the CIBERSORT only processes HUGO nomenclature in its pipeline. The analysis was performed using 100 permutations. The 22 immune cell types were later aggregated into a subset of distinct groups.

### Chromatin immunoprecipitation sequencing (ChIP-seq) assay and analysis

ChIP-seq was performed for H3K27me3 and H2A119ub1 with the protocol provided by Diagenode (iDeal ChIP-seq kit for histones; #C01010051). In brief, samples were cross-linked with formaldehyde for 10 min at room temperature and subsequently quenched with glycine. Samples were lysed and sonicated for at least 20 cycles of 30 s on and 30 s off using Diagenode Bioruptor Pico. For ChIP, 5 µg of antibody was conjugated with 50 µl of protein A magnetic beads. Immunoprecipitated DNA was processed for library preparation. Libraries were sequenced using the Illumina HiSeq2500 genome analyzer (65 bp, single end) and sequence reads were aligned to *Mus* *musculus* genome version 10 (mm10) using TopHat with the default setting. Reads with mapping quality >20 were selected, and peak calling over input control was performed using MACS2. Raw sequence data and bigWig data have been deposited in the Gene Expression Omnibus database (accession no. GSE145022).

### Drug sensitivity assay

The mesothelioma cell lines were seeded in 500 cells per well in a 384-well plate. The next day, drugs were added in a matrix format at the indicated concentration using the HP D300 digital dispenser (HP), and cells were grown in the presence of drug(s) or DMSO control for 5 d. Thereafter, 10% vol/vol Alamar blue was added to the well and incubated at 37°C for 4 h. The plates were read with the Tecan reader. The data were analyzed and plotted as drug response curves. For inhibition of PI3K, 20 nM BEZ-235 (concentration below IC50 dose) was used for 3 d in mesothelioma cells grown in 10-cm plates. All experiments were performed at least twice, and representative results are shown.

### Colony formation assay

For low-density colony formation, the mesothelioma cell lines were seeded in 5,000 cells per well in a 6-well plate and allowed to adhere overnight. The next day, drugs were added (at the indicated concentration), and cells were grown in the presence of drug or DMSO (control) for 7 d. At the end, plates were simultaneously fixed and stained with 6% glutaraldehyde with 0.1% crystal violet solution and digitized on an image scanner. All experiments were performed at least twice, and representative results are shown.

### Statistics

Data were statistically analyzed with GraphPad Prism software (version 7.0). P values were calculated by two-tailed *t *test or by Mann-Whitney test as specified in each figure legend. Survival curve P values were calculated by log-ranked Mantel-Cox test.

### Online supplemental material

Fig. S1 shows characterization of mouse models of mesothelioma. Fig. S2 presents characterization of oncogenic pathways and tumor microenvironment. Table S1 lists the differentially expressed genes, enriched KEGG pathway, IPA pathway, mice strain, and antibodies used in the study.

## Supplementary Material

Table S1 lists differentially expressed genes, KEGG pathways, mice strains, and antibodies.
